# Wie geht es den Menschen ab 80 Jahren in Deutschland gesundheitlich?

**DOI:** 10.1007/s00103-026-04287-7

**Published:** 2026-09-10

**Authors:** Judith Fuchs, Enno Nowossadeck, Anke-Christine Saß

**Affiliations:** Abteilung für Epidemiologie und Gesundheitsmonitoring, Robert Koch-Institut, Gerichtstr. 27, 13347 Berlin, Deutschland

Menschen in Deutschland und vielen anderen Ländern werden heute älter als in früheren Generationen. Damit gewinnt die Gesundheit von älteren und hochaltrigen Menschen immer mehr an Bedeutung. Die durchschnittliche Lebenserwartung bei Geburt liegt heute bei 83,4 Jahren für Frauen und 78,8 Jahren für Männer. Für 80-Jährige beträgt die fernere Lebenserwartung 9,7 Jahre bei Frauen und 8,2 Jahre bei Männern, das ist ein Anstieg um jeweils 1,8 Jahre in den letzten 30 Jahren. Die Veränderungen in der Lebenserwartung bringen individuell wie gesellschaftlich zahlreiche Chancen und Herausforderungen mit sich.

Im Jahr 2024 lebten nach Angaben des Statistischen Bundesamts 6,1 Mio. hochaltrige Menschen (ab 80 Jahren) in Deutschland; dies entsprach einem Anteil von 7,2 % an der Gesamtbevölkerung. Die Zahl der Hochaltrigen könnte laut Prognosen von Destatis in den nächsten 15 Jahren steigen, sodass sich ihr Anteil an der Gesamtbevölkerung auf rund 9 % belaufen würde. Angesichts dieser demografischen Veränderungen ist eine verstärkte wissenschaftliche Beschäftigung mit hochaltrigen Menschen, ihren individuellen Problemlagen und Lebenswelten erforderlich. Sie bildet die Grundlage für evidenzbasierte Maßnahmen in Prävention und Versorgung sowie für die Gestaltung gesundheitsförderlicher Lebenswelten.

Auch die Weltgesundheitsorganisation (WHO) hebt hervor, dass der demografische Wandel zahlreiche Anforderungen für Individuum und Gesellschaft mit sich bringt. Ein vielversprechender Ansatz, um Gesundheit, Selbstständigkeit und Teilhabe im höheren Alter zu fördern, ist das Konzept des „Healthy Ageing“. Es geht davon aus, dass die gesundheitliche Lage älterer Menschen sowohl durch ihre körperlichen und geistigen Fähigkeiten als auch ihr Lebensumfeld bestimmt ist. Die WHO hat sich für die aktuelle Dekade des gesunden Alterns (2021–2030) zum Ziel gesetzt, die Lebensqualität älterer Menschen, ihrer Familien sowie der Gemeinschaften, in denen sie leben, zu verbessern.

Die gesundheitliche Lage hochaltriger Menschen gewinnt an Bedeutung, da mit zunehmendem Alter Demenz, Multimorbidität, Gebrechlichkeit und Pflegebedürftigkeit sowie Erkrankungen wie Herz-Kreislauf-Erkrankungen, Typ-2-Diabetes und Arthrose gehäuft auftreten und einen wachsenden Versorgungsbedarf nach sich ziehen. Im 9. Altenbericht der Bundesregierung wird ausgeführt, dass insbesondere die medizinische und pflegerische Versorgung hochaltriger Menschen eine zunehmende Herausforderung darstellt. Zugleich gibt es zahlreiche Erkenntnisse darüber, wie gesundes Altern gelingen kann. Entscheidend ist dabei nicht nur, Krankheiten zu vermeiden oder ihre Folgen zu begrenzen, sondern auch das Lebensumfeld so zu gestalten, dass Lebensqualität und Autonomie erhalten oder verbessert werden. Inwieweit diese Erkenntnisse für hochaltrige Menschen in Deutschland umgesetzt werden, wurde bislang nicht zusammenfassend dargestellt.

Das Themenheft hat das Ziel, relevante Handlungsfelder für die Gesundheit hochaltriger Menschen und die Gestaltung ihrer Lebenswelten zu beschreiben sowie vielversprechende Ansatzpunkte aufzuzeigen, um gesundheitliche Ressourcen auf individueller Ebene und im Lebensumfeld zu stärken. Die 9 Original- und Übersichtsarbeiten sind 3 Themenkomplexen zugeordnet.

Das Heft beginnt mit einem Überblick über die gesundheitliche Lage hochaltriger Menschen. *Wolter et al.* beschreiben demografische Eckdaten der Menschen ab 80 Jahren in Deutschland. Sie berichten über ihre soziale Lage und gesundheitliche Herausforderungen, auch mit Blick auf regionale Unterschiede, und gehen auf die künftige Entwicklung des Bevölkerungsanteils hochaltriger Menschen ein.

Der 2. Themenkomplex umfasst 5 Beiträge, die sich mit gesundheitsbezogenen Ressourcen und Risiken hochaltriger Menschen auf individueller Ebene beschäftigen. *Nowossadeck et al.* widmen sich den körperlichen Einschränkungen hochaltriger Menschen und berücksichtigen sowohl übergreifende Maße für Einschränkungen bei Alltagsaktivitäten als auch spezifische Indikatoren wie Mobilitäts‑, Seh- und Höreinschränkungen, Harninkontinenz und Gebrechlichkeit (Frailty), die für Teilhabe und Autonomie hochrelevant sind. *Wittmann et al.* beschäftigen sich mit Risiko- und Schutzfaktoren für Demenz. Sie geben einen Überblick über den aktuellen Forschungsstand und die bekannten Risikofaktoren. Darüber hinaus berücksichtigen sie bislang weniger beachtete Einflussfaktoren, stellen bestehende Interventionsansätze vor und diskutieren deren gesundheitspolitische Relevanz. *Fuchs et al.* analysieren auf Basis einer aktuellen Studie das Sturzgeschehen, das angesichts des wachsenden Anteils hochaltriger Menschen zunehmend an Bedeutung gewinnt. Sie zeigen zudem auf, welche Risikofaktoren mit Stürzen assoziiert sind. Ein besonderer Schwerpunkt liegt auf der Frage, inwieweit hochaltrige Menschen Medikamente einnehmen, die das Sturzrisiko erhöhen können. *Hoffmann et al.* beschreiben auf Basis ihres narrativen Reviews die gesundheitliche Lage von Menschen in stationären Langzeitpflegeeinrichtungen. Sie fassen Ergebnisse aus den letzten 10 Jahren zusammen und zeigen Datenlücken auf. *Hajek und König *untersuchen anhand des Deutschen Alterssurveys Zusammenhänge zwischen der Beziehungsqualität zu Familie und Freunden, depressiven Symptomen und Einsamkeit. Sie zeigen, dass eine hohe Beziehungsqualität zu Freunden bei hochaltrigen Menschen mit weniger depressiven Symptomen und geringerer Einsamkeit assoziiert ist.

Im 3. Themenkomplex beschäftigen sich 3 Beiträge mit den Lebenswelten hochaltriger Menschen. Der Überblicksbeitrag von *Höppner und Brandt* thematisiert die mit dem Klimawandel verbundenen Herausforderungen für hochaltrige Menschen – angesichts zunehmender Hitzeperioden ein hochaktuelles Thema. Sie beleuchten ein neues Forschungsfeld und verdeutlichen zugleich, dass hochaltrige Menschen eine heterogene Gruppe sind, woraus sich Implikationen für die künftige Forschung ergeben. *Koller et al. *setzen sich mit dem Konzept der Walkability, also der „Gehfreundlichkeit“ der Umgebung hochaltriger Menschen, auseinander und zeigen, dass bisherige Konzepte deren besondere Bedürfnisse nicht hinreichend abbilden. Sie stellen ein Rahmenkonzept und ein Anwendungsbeispiel mit altersgerechten Dimensionen vor. *Blotenberg und Gebert* beschäftigen sich mit dem häuslichen Wohnumfeld älterer Menschen und beschreiben Präventive Hausbesuche, das heißt aufsuchende Beratungs- und Unterstützungsangebote in den Kommunen. Sie machen auf die Heterogenität der Angebote aufmerksam, die eine Überführung in ein Regelangebot der kommunalen Seniorenarbeit erschweren kann.

Die Beiträge des vorliegenden Themenheftes machen deutlich, dass wir heute über ein breites Wissen zur gesundheitlichen Lage und zu den gesundheitsbezogenen Lebenswelten der Menschen ab 80 Jahren verfügen. Gleichzeitig zeigen sie konkrete Ansatzpunkte auf, um Gesundheit, Selbstständigkeit und Teilhabe auch im sehr hohen Alter zu fördern. Angesichts der wachsenden Zahl hochaltriger Menschen gilt es, diese Erkenntnisse im Sinne von „Health in All Policies“ konsequent in politische Entscheidungen und die Versorgungspraxis zu überführen. Dazu zählen altersgerechte und gesundheitsförderliche Lebenswelten, die ein selbstbestimmtes Leben auch im hohen Alter ermöglichen, die Stärkung von Verhaltens- und Verhältnisprävention und Gesundheitsförderung, die Förderung sozialer Teilhabe sowie der sinnvolle Einsatz technologischer Innovationen. Die Herausforderungen sind bekannt – nun kommt es darauf an, das vorhandene Wissen entschlossen in konkrete Maßnahmen zu überführen.

Wir wünschen Ihnen eine interessante und inspirierende Lektüre!

## Data Availability

Alle dieser Arbeit zugrunde liegenden Daten sind in diesem Artikel enthalten.

